# Understanding the Effect of Introducing Micro- and Nanoparticle Bismuth Oxide (Bi_2_O_3_) on the Gamma Ray Shielding Performance of Novel Concrete

**DOI:** 10.3390/ma14216487

**Published:** 2021-10-28

**Authors:** Mohamed A. El-Nahal, Mohamed Elsafi, M. I. Sayyed, Mayeen Uddin Khandaker, Hamid Osman, Basem H. Elesawy, Ibrahim H. Saleh, Mahmoud I. Abbas

**Affiliations:** 1 Department of Environmental Studies, Institute of Graduate Studies and Research, Alexandria University, Alexandria 21526, Egypt; Igsr.nahalmoh@alexu.edu.eg (M.A.E.-N.); igsr.ihindawy@alexu.edu.eg (I.H.S.); 2 Physics Department, Faculty of Science, Alexandria University, Alexandria 21511, Egypt; mabbas@physicist.net; 3 Department of Physics, Faculty of Science, Isra University, Amman 11622, Jordan; dr.mabualssayed@gmail.com; 4 Department of Nuclear Medicine Research, Institute for Research and Medical Consultations (IRMC), Imam Abdulrahman Bin Faisal University (IAU), P.O. Box 1982, Dammam 31441, Saudi Arabia; 5 Centre for Applied Physics and Radiation Technologies, School of Engineering and Technology, Sunway University, Bandar Sunway 47500, Selangor, Malaysia; mayeenk@sunway.edu.my; 6 Department of Radiological Sciences, College of Applied Medical Sciences, Taif University, Taif 21944, Saudi Arabia; ha.osman@tu.edu.sa; 7 Department of Pathology, College of Medicine, Taif University, P.O. Box 11099, Taif 21944, Saudi Arabia; b.elesawy@tu.edu.sa

**Keywords:** concrete, bulk Bi_2_O_3_, nanoparticle Bi_2_O_3_, MAC, HVL, RPE, LAC

## Abstract

The aim of this study is to investigate the radiation shielding properties of novel concrete samples with bulk Bi_2_O_3_ and Bi_2_O_3_ nanoparticles (Bi_2_O_3_ NP) incorporated into its composition. The mass attenuation coefficient of the concrete samples without Bi_2_O_3_ and with 5 and 7 wt% bulk Bi_2_O_3_ were experimentally determined and were compared against values obtained using the XCOM and Geant4 simulations. Both methods greatly agree with the experimental values. The linear attenuation coefficients (LAC) of blank concrete (C-0), concrete with 5% bulk Bi_2_O_3_ (C-B5), and concrete with 5% nanoparticle Bi_2_O_3_ (C-N5) were determined and compared at a wide energy range. We found that the LAC follows the trend of C-0 < C-B5 < C-N5 at all the tested energies. Since both C-B5 and C-N5 have a greater LAC than C-0, these results indicate that the addition of Bi_2_O_3_ improves the shielding ability of the concretes. In addition, we investigated the influence of nanoparticle Bi_2_O_3_ on the LAC of the concretes. The half-value layer (HVL) for the concretes with bulk Bi_2_O_3_ and Bi_2_O_3_ nanoparticles is also investigated. At all energies, the C-0 has the greatest HVL, while C-N15 has the least. Thus, C-N15 concrete is the most space efficient, while C-0 is the least space efficient. The radiation protection efficiency (RPE) of the prepared concretes was found to decrease with increasing energy for all five samples. For C-0, the RPE decreased from 63.3% at 0.060 MeV to 13.48% at 1.408 MeV, while for C-N15, the RPE decreased from 87.9 to 15.09% for the same respective energies. Additionally, C-N5 had a greater RPE than C-B5, this result demonstrates that Bi_2_O_3_ NP are more efficient at shielding radiation than bulk Bi_2_O_3_.

## 1. Introduction

Radiation shielding tools have become increasingly more important in modern society due to the vast spread of radioactive sources in various fields of work. Workers and patients who come in contact with radiation for long periods of time are at risk of exposure to nuclear radiation, which can severely impact their health. Therefore, it is necessary to protect these people from the risks of ionizing radiation. The type of protection tools and materials chosen for a specific application depends upon several factors, which include the intensity of the photons and the energy of the radiation source. Additionally, the method used will vary depending upon the economic preparation of the materials and the resistance of the material to radiation damage [1,2]. Concrete is an effective shielding material that has gained much interest due to its low cost, eco-friendliness, suitable density for radiation attenuation, and simplicity to manufacture when comparing with other traditional shielding materials [3,4]. Furthermore, it is simple to incorporate high atomic number elements in the concrete in order to improve its attenuation capability [5]. In addition to these advantages, concrete can be molded into several shapes, requires low maintenance, and has good mechanical features. All of the aforementioned characteristics make concrete a versatile and attractive material to be used in the radiation shielding field [6,7,8]. In the past, different concretes consisting of specific aggregates and ingredients to improve its radiation shielding features have been fabricated. Madej et al. [9] prepared refractory concretes containing new types of cements and investigated their radiation protection performance. They reported the linear attenuation coefficients in the range of 80–1408 keV. Prochon and Piotrowski [10] reported the effect of cement and aggregate-type on the radiation protection performance of a certain kind of concrete using Monte Carlo simulation method. Lotfi-Omran et al. [11] investigated the influence of cement content and maximum aggregate size on the radiation shielding features of heavyweight concrete. Lotfi-Omran et al. [12] explored the role of different water to cement ratio on the mechanical properties and radiation protection efficiency of heavyweight magnetite concrete. For this purpose, they used ^137^Cs and ^60^Co sources, and they found that the radiation protection efficiency is enhanced with the reduction of the water-to-cement ratio. Demir et al. [13] investigated the role of high temperatures on the radiation attenuation parameters for polypropylene fiber-reinforced heavyweight concrete. Sikora et al. [14] evaluated the effect of Bi_2_O_3_ in both sizes (micro- and nano-size) on the radiation shielding performance of Portland cement pastes. The authors concluded that the addition of micro- and nano-Bi_2_O_3_ is an alternative method to produce lead-free radiation shielding materials.

Most of the previous works focused on studying the influence of the energy, the density of the concretes, and the types of aggregates added to the radiation attenuation ability of the radiation shields.

By introducing nanoparticles into the composition of concretes, the radiation shielding properties of concrete can be enhanced, as well as its structural and mechanical properties. Nanoparticles improve these characteristics by improving the bulk properties or packing model structure of the concrete samples [14,15]. These particles can act as fillers that make the concrete more compact and increase their density. Nanoparticles (NPs) can also eliminate small pores and deterioration in the structure by acting as a filler. Additionally, some NPs are able to act as binding agents that are smaller than cement particles, enhancing the structure of the concrete. These resulting concretes have been prepared in previous studies and have demonstrated to be more durable and efficient than ordinary concrete. NPs are becoming especially prominent in the production of ultra-high performance concrete (UHPC) as a substitute to silica fume, which is limited and has a high cost. This nano-silica is currently being used in many nano-processes that involve concrete. Other nanoparticles that are introduced into concrete include alumina, titanium oxide, carbon nanotubes, and polycarboxylates [16,17].

With respect to radiation shielding performance, nanoparticles are more effective than larger micro-particles due to the greater surface-area-to-volume ratio achieved by the NPs, which results in greater attenuation. This result occurs because of the more homogenous distribution caused by NPs, increasing particle density and lowering the grain size inside the material matrix. Although these effects have been postulated, these improvements are often only seen at low energies (<0.03 MeV), while at higher energies, the effect of particle size on the radiation shielding ability of the samples is minimal [18,19,20].

There is currently limited knowledge on the effect of incorporating Bi_2_O_3_ nanoparticles into cement-based composites. Within the studies that use Bi_2_O_3_ NPs, most of them evaluate the gamma-ray attenuation abilities of the Bi_2_O_3_ NP concretes, but none have tested their abilities against neutron radiation. Thus, more research is needed to understand the effect of Bi_2_O_3_ NPs content and size on the structural, mechanical, and radiation shielding properties of concrete.

In this work, the MAC of the concrete samples without Bi_2_O_3_ and with 5 and 7 wt% bulk Bi_2_O_3_ were experimentally determined and were compared against values obtained using the XCOM and Geant4 simulations. The LAC of blank concrete (C-0), concrete with 5% bulk Bi_2_O_3_, and concrete with 5% nanoparticle Bi_2_O_3_ were determined and compared at a wide energy range. Other shielding parameters were investigated, such as half-value layer (HVL), mean free pass (MFP), tenth value layer (TVL), and radiation protection efficiency (RPE). All of these parameters indicated that the radiation shielding for concrete improved better with bismuth nanoparticles than bismuth micro.

## 2. Materials and Methods

### 2.1. Samples Preparation

Two sets of concrete samples were developed according to the method described by Basu et al. [21]. The mixing ingredient ratio was 1:1.5:3:0.5 of cement, fine aggregates, coarse aggregates and water, respectively. The first sample set contained bulk bismuth oxide with concentration of 5 and 7% of weight of the fine aggregate of the concrete sample. The other set included three concentrations of nano-bismuth oxide 5, 10 and 15% of fine aggregate weight. The bismuth oxide (cornel chemical laboratory company, Cairo, Egypt) was considered as a partial substitution of fine aggregates. All samples were prepared identically from Portland cement (Alexandria Cement Company, Alexandria, Egypt), sand as fine aggregates, and marble Egyptian Galala, which is the most available type of marble in Egypt as coarse aggregates. The volume and particle size of coarse aggregates were as reduced as possible to ensure the homogeneity of the mixture; bismuth oxide was mixed with sand first to form fine aggregates, which were mixed with the coarse aggregates in the mixer until forming a homogenous mix. Then, the addition of cement was followed by water to create the sample; water to cement ratio “W/C” was kept constant in order to estimate the samples of different capabilities of gamma attenuation.

The samples included fine aggregate composition with bulk bismuth oxide concentration of more than 7%, which were not succeeded. Concentrations greater than 15% of the nanoparticle bismuth oxide could not be developed. All rejected samples exhibited low compactness. The mixture composition of each sample is shown in Table 1. Concrete sample without adding bismuth oxide was also prepared and investigated. Prepared samples were poured into cylindrical molds with dimensions of 4 × 16 cm^2^ after mixing, and then they were cut into slabs of 4 × 1 cm^2^ to be measured through the narrow beam attenuation method.

### 2.2. Samples Characterization

**Figure 1:**
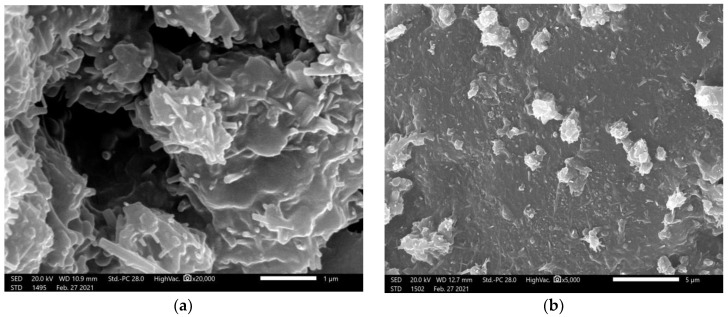

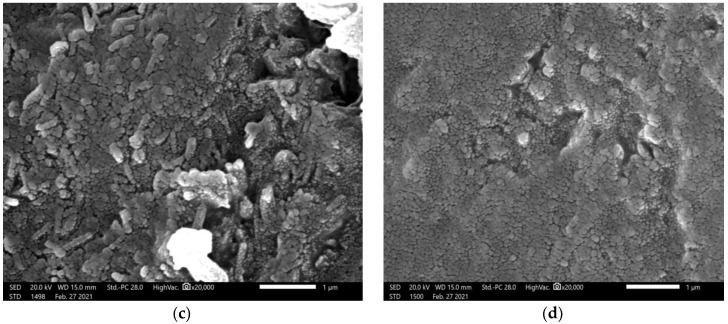
The scanning microscope images of (**a**) blank concrete sample, (**b**) concrete sample with bulk 5% of bismuth oxide sample, (**c**) concrete sample with 5% of nano-bismuth oxide sample, and (**d**) concrete sample with 10% of nano-bismuth oxide sample.

The elemental compositions of producing samples were determined by using energy dispersive X-ray analysis EDX unit the of electron scanning microscope. The sample compositions are given in Table 2. Water content remaining inside the samples was estimated according to the method of Piotrowskia et al. [22]. Scanning electron microscope (JSM-5300, JEOL, Tokyo, Japan) was utilized to assess the homogeneity of the distribution of micro-bismuth oxide and nano-bismuth oxide inside the sample and to determine the size of bismuth oxide in the samples as shown in Figure 1. The samples were fixed with double-coated carbon tap, which also dissipated the electron beam charge and heat buildup. Samples were covered with a fine layer of gold under vacuum before SEM observation, using an ion sputtering coating device (JEOL-JFC-1100E). They were operated at 25 kV at a magnification order of 35,000.

### 2.3. Geant4 Code

**Figure 2:**
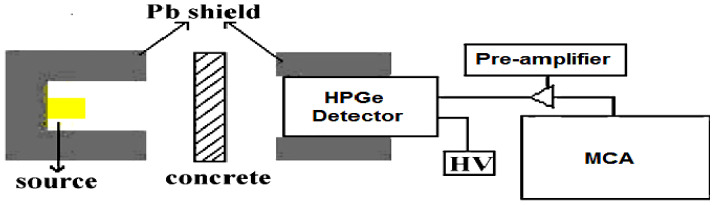
The schematic diagram of experimental setup of narrow beam method.

To reinforce this article, Monte Carlo Geant4 Simulation version 10.3.P3 was used. In the present work, the detector simulated at the x-plane with the same condition in the experimental work. At the top of the detector, the absorbed concrete sample was simulated. The thickness of the concrete sample was optimized according to the energy of the falling beam to avoid all photons being absorbed into the concrete sample or traversing the sample plate without reaction. The monoenergetic photons were simulated in this work to avoid the coincidence summing effect as well as to give us a broad range of energy from 0.015 up to 15 MeV [23,24,25,26]. The physical processes were considered, including photoelectric, Compton, and pair production interactions. The incident beam was designed as the narrow beam by using a lead collimator, as shown in Figure 2.

**Figure 3:**
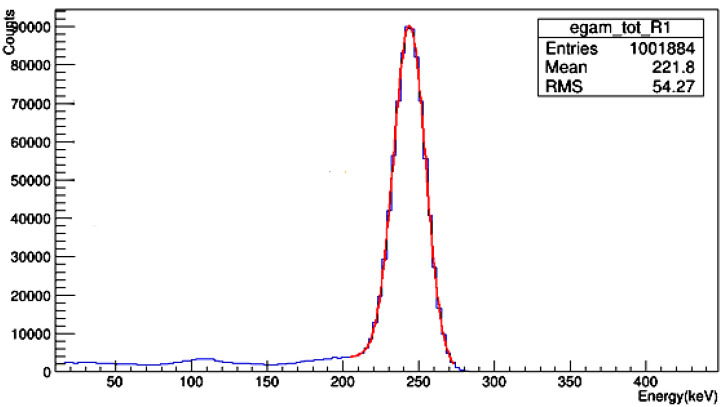
The simulated spectrum at 0.245 MeV using Geant4 code, where the blue spectrum is the original spectrum and the red spectrum due to the Gaussian fitted spectrum.

The run of this simulation gave the spectrum using ROOT software [27,28] as shown in Figure 3. From the spectrum, the area under the peak or the counts can be calculated, which represent the intensity of the line corresponding to this area. Thus, the MAC was evaluated using the calculated area in the presence of the concrete sample (I) and in the absence sample (I_0_) with the same simulated primary photons and energy as the following equation [29]:

(1) $$\mathrm{MAC}=-\frac{\ln(\frac{I}{{\;I}_{0}})}{x\times \rho }$$

In the previous equation, *x* (cm) is the thickness of the concrete sample, and *ρ* (g/cm^3^) is its density.

### 2.4. Attenuation Measurement

Samples attenuation parameters were determined by the narrow beam method [30]; the collimated beam of gamma radiation with different energies were transmitted through the samples. The transmitted radiation was measured by using Canberra High Purity Germanium gamma ray spectrometer (HPGe) of the model: CS20-A31CL, equipped with multichannel analyzer (MCA). The detector relative efficiency was 24.5% for 1.333 MeV of Co-60-line relation to 3 × 3 in^2^ NaI scintillation detector and 25 cm source detector distance. The specifications of radiation sources used in this experiment in order to emit gamma radiation with various energies are given in the Table 3. The experiment set up is shown in Figure 2. The initial and transmitted intensities of each considered gamma line were determined for a fixed counting time by integrating the area under the photo peak, which represents the intensity of gamma rays. The counting time was selected to be large enough to obtain statistical uncertainty below 1%. The spectrum was analyzed by using Genie 2000 data acquisition and analysis software, which is comprehensive software for acquiring and analyzing gamma ray spectra. The detector energy and efficiency calibrations were performed prior to the measurement by utilizing three radioactive sources Am-241 (0.595 MeV), Cs-137 (0.661 MeV) and Co-60 (1.173 and 1.332 MeV) to cover the experimental measurement scale of energy [31,32].

### 2.5. Shielding Parameters:

MAC’s were estimated theoretically using the “mixture rule” and the XCOM computer software developed by NIST. The mixture can be expressed by the following relation [29]:

MAC = Ʃ w_i_ (MAC)_i_ (2)

where w_i_ and (MAC)_i_ are weight fractions and the MAC of the constituent elements. Experimental MAC was calculated using Equation (1). By multiplying the mass attenuation coefficient in the density *ρ* (g/cm^3^), we obtain an essential parameter called the linear attenuation coefficient or LAC (cm^−1^), which measures the probability of photon interaction inside the material per unit path length. The half-value layer (HVL) and the tenth-value layer (TVL) values are important attenuating factors and are defined as the thickness needed to reduce the intensity of incident photon to half- and tenth- initial values, respectively, and can be calculated using the following relations [33].

(3) $$\mathrm{HVL}=\frac{\mathrm{Ln}2}{\mathrm{LAC}}$$

(4) $$\mathrm{TVL}=\frac{\mathrm{Ln}10\;}{\mathrm{LAC}}$$

The mean-free path or MFP (cm) is the reciprocal of attenuation coefficient and is also defined as the average distance between two successive interactions of gamma rays inside the sample material, which can be estimated by Equation (5).

(5) $$\mathrm{MFP}=\frac{1}{\mathrm{LAC}}$$

The shielding efficiency of an absorber sample can be investigated using a parameter called the radiation protection efficiency (RPE), which depends on the net count rate (intensity) with and without the absorber during the measurement and is given by Equation [34].

(6) $$\mathrm{RPE}=(1\;-\;\frac{I}{I_{0}})\;\times \;100$$

## 3. Results and Discussion

The mass attenuation coefficient of the concrete samples (C-0, C-B5, and C-B7) was experimentally determined and was compared against values obtained using the XCOM and Geant4 simulations (see Table 4). First, the relative error between the experimental method and the XCOM simulation was calculated and is represented by ∆_1_% and given by:

(7) $$∆1\left(\%\right)=\left(\left[{\left(\mathrm{MAC}\right)}_{\mathrm{XCOM}}-{\left(\mathrm{MAC}\right)}_{\mathrm{EXP}}\right]÷{\left(\mathrm{MAC}\right)}_{\mathrm{EXP}}\right)\times 100$$

All of these values are within less than ±2% deviation, with the greatest difference equal to −1.97% and the least to 0.19%. These results indicate that the experimental and XCOM results strongly agree with each other, confirming the accuracy of the experimental values. Additionally, the percent difference between the experimental and the Geant4 simulation was also calculated and is represented by ∆_2_% and given by:

(8) $$∆2\left(\%\right)=\left(\left[{\left(\mathrm{MAC}\right)}_{\mathrm{Geant}4}-{\left(\mathrm{MAC}\right)}_{\mathrm{EXP}}\right]÷{\left(\mathrm{MAC}\right)}_{\mathrm{EXP}}\right)\times 100$$

For these values, all the results were within a deviation of less than ±1.2%, which is closer than the XCOM values, indicating that the Geant4 values agree with the experimental values more than the XCOM results. Nevertheless, both methods greatly agree with the experimental values. In addition, the MAC values can be analyzed against increasing energy. In this case, all the values decrease with increasing energy. For instance, for the C-0 sample, its experimental MAC values decrease from 0.3754 cm^2^/g at 0.060 MeV to 0.1194 cm^2^/g at 0.245 MeV, 0.0765 cm^2^/g at 0.662 MeV, 0.0585 cm^2^/g at 1.173 MeV, and 0.0541 cm^2^/g at 1.408 MeV. This decrease occurs since higher energy photons have an easier time penetrating through the samples, decreasing MAC. The MAC values of the samples can also be compared against each other. At all energies, the MAC results follow the order of C-0 < C-B5 < C-B7. More specifically, at 0.344 MeV, for example, the MAC values are equal to 0.1016, 0.1068, and 0.1049 cm^2^/g for C-0, C-B5, and C-B7, respectively. These results indicate that the C-B7 sample has the greatest MAC out of these three samples, meaning it has the best shielding ability at all the tested energies.

**Figure 4:**
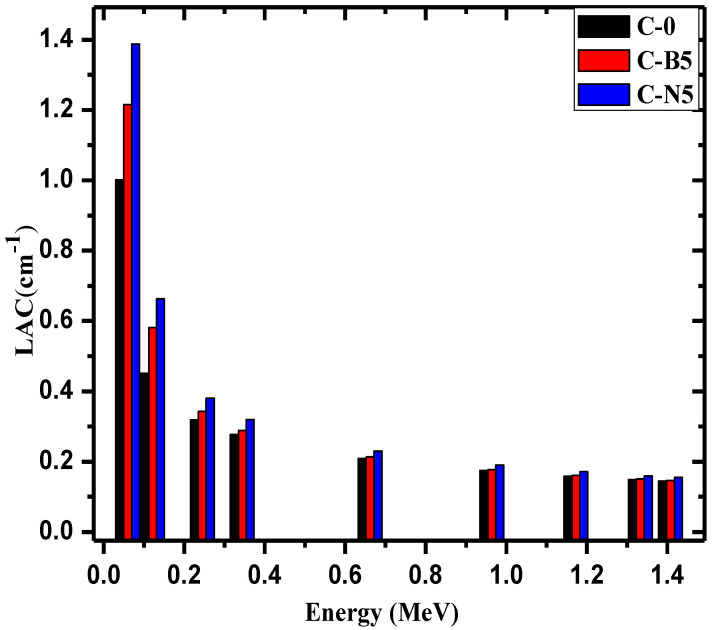
The linear attenuation coefficient for the C-0, C-B5 and C-N5.

The linear attenuation coefficients (LAC) of blank concrete (C-0), concrete with 5% bulk Bi_2_O_3_, and concrete with 5% nanoparticle Bi_2_O_3_ were determined at a wide energy range and plotted in Figure 4. When observing this figure, it can be seen that the LAC follows the trend of C-0 < C-B5 < C-N5 at all the tested energies. At 0.662 MeV, for example, the LAC values are equal to 0.209 cm^−1^ for C-0, 0.213 cm^−1^, and 0.230 cm^−1^ for C-N5. Since both C-B5 and C-N5 have a greater LAC than C-0, these results indicate the addition of Bi_2_O_3_ improves the shielding ability of the concretes. It is well known that the LAC has a direct relation with the density of the medium [35]. Additionally, the results indicate that the C-N5 concrete has the best shielding ability out of these three samples, as it has the greatest LAC at all tested energies.

**Figure 5:**
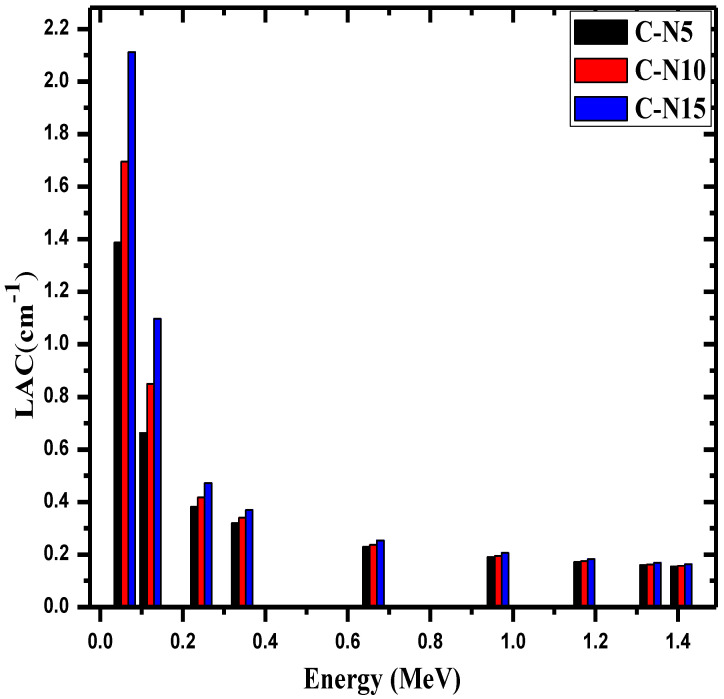
The linear attenuation coefficient for the C-N5, C-N10 and C-N15.

Figure 5 analyzes the influence of nanoparticle Bi_2_O_3_ on the LAC of the concretes. Samples with 5, 10, and 15% Bi_2_O_3_ were selected to observe the effect of increasing the nanoparticle Bi_2_O_3_ concentration on the shielding ability of the concretes. At all energies, the LAC values have the trend of C-N5 < C-N10 < C-N15. At 0.245 MeV, for instance, the LAC values increased from 0.381 to 0.418 and 0.472 cm^−1^ for C-N5, C-N10, and C-N15, respectively. These results demonstrate that increasing the amount of nanoparticle Bi_2_O_3_ improves the shielding ability of the concrete. Furthermore, the influence of Bi_2_O_3_ on the LAC values is clearer at low energies. For example, at 0.0595 MeV, C-N5′s LAC was 1.39 cm^−1^, C-N10′s LAC was 1.70 cm^−1^, and C-N15′s LAC was 2.11 cm^−1^. This trend can be understood according to the domination of photoelectric process. At higher energies, meanwhile, the effect of Bi_2_O_3_ on the shielding ability of the concretes is minimal, and the difference between their capabilities of the samples is small. This is a result of the domination of Compton scattering which has a weak dependence on the chemical composition of the medium. The LAC values at 1.41 MeV are equal to 0.155, 0.158, and 0.164 cm^−1^ for C-N5, C-N10, and C-N15, respectively. Nevertheless, at all energies, the C-N15 sample had the greatest LAC out of these three samples.

**Figure 6:**
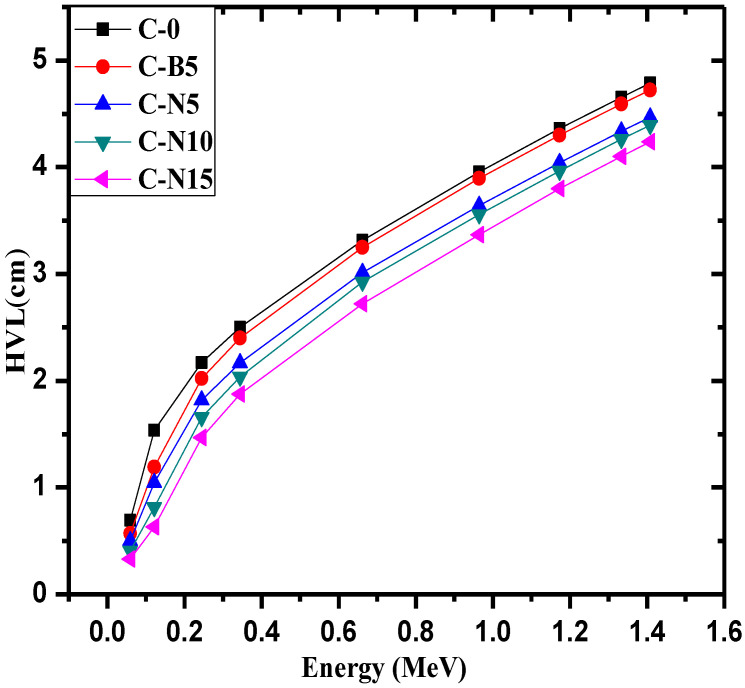
The half-value layer for the C-0, C-B5, C-N5, C-N10 and C-N15.

The half-value layer (HVL) is an important radiation shielding parameter [36,37,38,39]. The HVL for C-0, C-B5, C-N5, C-N10, and C-N15 is illustrated in Figure 6. At all energies, the C-0 has the greatest HVL at all energies, while C-N15 has the least. For example, at 0.0595 MeV, C-0 has an HVL of 0.683 cm while C-N15 has an HVL of 0.328 cm, while at 1.41 MeV. the HVL values are equal to 4.68 and 4.24 cm for C-0 and C-N15, respectively. Since the C-N15 sample has the least HVL at all energies, this concrete is the most space efficient, while C-0 is the least space efficient. Moreover, when comparing C-B5 and C-N5, which both have the weight fraction of Bi_2_O_3_, it can be seen that the HVL for the concrete with NP Bi_2_O_3_ is lower than that of bulk Bi_2_O_3_, which means that the use of NPs decreases the thickness of the concretes and improves their ability to shield photons.

**Figure 7:**
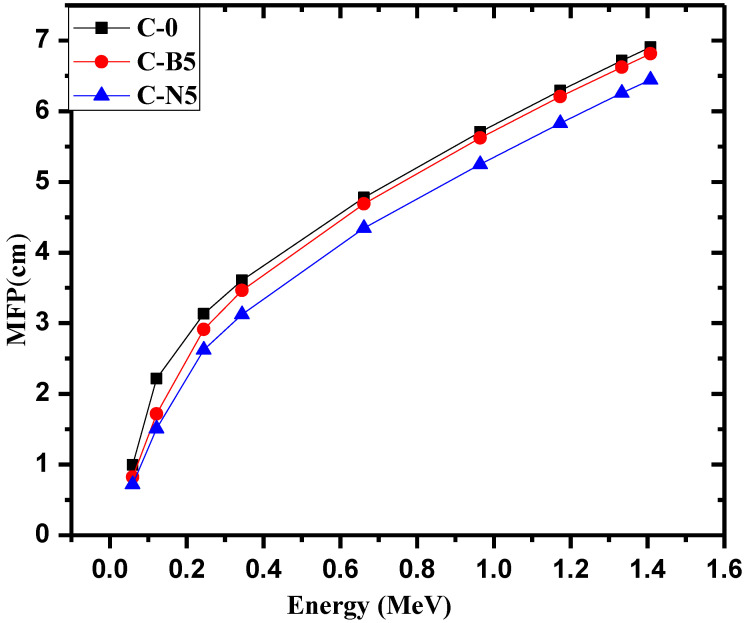
The mean free path for the C-0, C-B5 andC-N5.

The mean free path (MFP) for the C-0, C-B5, and C-N5 concretes are illustrated against increasing energy in Figure 7. At all energies, MFP increases with increasing energy. This means that the ability of the photons to penetrate the samples under study increases with increasing the energy. The MFP of the C-0, for example, increases from 0.986 cm at 0.0595 MeV to 3.06 cm 0.245 MeV, 4.78 cm at 0.662 MeV, 6.25 cm at 1.17 MeV, and 6.76 cm at 1.41 MeV. For the C-N5 sample, the MFP is equal to 0.721, 2.62, 4.35, 5.83, and 6.44 cm for the same respective energies. This increasing trend occurs because higher energy photons have an easier time penetrating through the samples, decreasing the number of collisions, and increasing the distance between collisions, increasing MFP. Another trend can be observed when analyzing the values at a single energy. At 0.662 MeV, for example, the MFP values are equal to 4.78, 5.07, and 5.25 cm for C-0, C-B5, and C-N5, respectively. This trend occurs at all energies and demonstrates that the C-N5 sample has the best shielding ability out of these three concretes.

**Figure 8:**
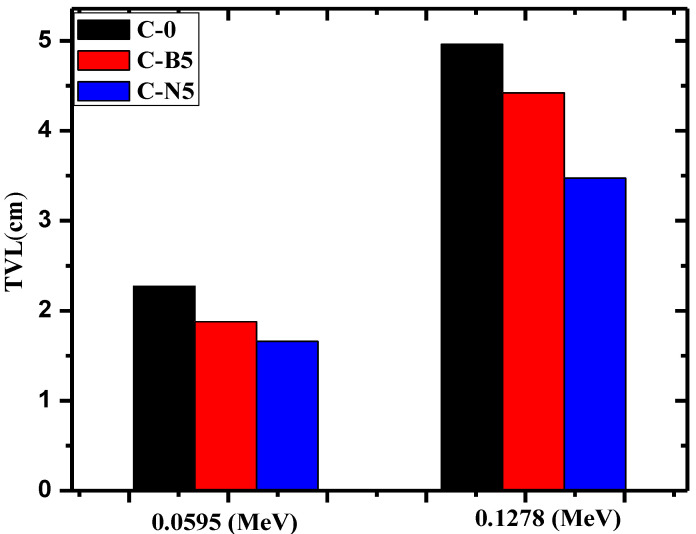
The tenth-value layer for the C-0, C-B5 and C-N5, at 0.059 and 0.1278 MeV.

Figure 8 graphs the tenth-value layer (TVL) at 0.0595 and 0.1278 MeV for C-0, C-B5, and C-N5. At both 0.0595 and 0.1278 MeV, the TVL follows the order of C-0 > C-B5 > C-N5. More specifically, at 0.0595 MeV, they are equal to 2.27, 1.88, and 1.66 cm for C-0, C-B5, C-N5, respectively, and at 0.1278 MeV, they are equal to 4.96, 4.42, and 3.47 cm for the same respective concretes. These results show that the TVL for blank concrete is higher than for 5% bulk Bi_2_O_3_ and 5% NP Bi_2_O_3_, with the NP Bi_2_O_3_ having the lowest TVL at both energies. It was also found that this trend is maintained at all the tested energies; thus, only these two energies were graphed. Figure 8 also shows that TVL increases with increasing energy, which agrees with the trend in the HVL figure.

**Figure 9:**
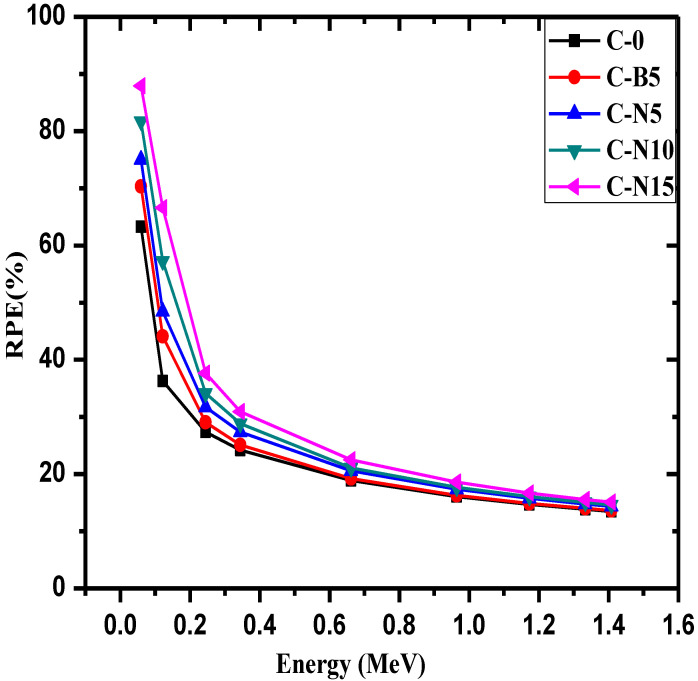
The radiation protection efficiency (RPE) of the prepared concretes.

The radiation protection efficiency (RPE) of the prepared concretes were graphed against increasing energy in Figure 9. First, it can be seen that RPE decreases with increasing energy for all five samples. For example, C-0′s RPE decreases from 63.3% at 0.060 MeV to 13.48% at 1.408 MeV and C-N15′s RPE decreases from 87.9 to 15.09% for the same respective energies. This decreasing trend occurs due to the increased penetration power of higher energy photons, which decreases the capability for these concretes to shield the incoming radiation. When comparing the values against each other, it can be seen that the RPE results follow the order of C-0 < C-B5 < C-N5 < C-N10 < C-N15. At 0.344 MeV, for example, the RPE values are equal to 24.41, 25.08, 27.39, 28.84, and 30.93% for C-0, C-B5, C-N5, C-N10, and C-N15, respectively. These results indicate that adding Bi_2_O_3_ to the concretes improve their shielding ability. Additionally, C-N5 has a greater RPE than C-B5, this result demonstrates that Bi_2_O_3_ NP are more efficient at shielding radiation than bulk Bi_2_O_3_. As the Bi_2_O_3_ further increases, the RPE values increase with it, meaning that increasing the Bi_2_O_3_ content in the concrete enhances the attenuation capability of the samples.

## 4. Conclusions

In the present work, the MAC of the concrete samples without Bi_2_O_3_ and with 5 and 7 wt% bulk Bi_2_O_3_ were experimentally determined and were compared against values obtained using the XCOM and Geant4 simulations. Both techniques greatly agree with the experimental results, implying the accuracy in the setup used for the determination of the MAC and LAC for the fabricated concretes. The LAC of blank concrete (C-0), concrete with 5% bulk Bi_2_O_3_, and concrete with 5% nanoparticle Bi_2_O_3_ were determined and compared at a wide energy range. From the obtained results, the following conclusions can be drawn:

1. The LAC results demonstrated that increasing the amount of nanoparticle Bi_2_O_3_ improves the shielding ability of the concrete since the LAC values have the trend of C-N5 < C-N10 < C-N15.
2. At all energies, the C-0 has the greatest HVL, while C-N15 has the least. Thus, C-N15 concrete is the most space efficient, while C-0 is the least space efficient.
3. The MFP is evaluated for C-0, C-B5, and C-N5 concretes and the MFP values at 0.662 MeV are equal to 4.78, 5.07, and 5.25 cm for C-0, C-B5, and C-N5, respectively, which demonstrated that the C-N5 sample has the best shielding ability out of these three concretes.
4. The radiation protection efficiency (RPE) of the prepared concretes was found to decrease with increasing energy for all five samples.
5. The RPE results revealed that increasing the energy of the photons leads to decreased capability for these concretes to shield the incoming radiation. Finally, C-N5 has a greater RPE than C-B5; this result demonstrates that Bi_2_O_3_ NP are more efficient at shielding radiation than bulk Bi_2_O_3_.

## Figures and Tables

**Table 1:** The concrete sample mixes composition.

Concrete Sample Code	Water Dosage, kg/m^3^	Cement Dosage, kg/m^3^	Fine Aggregate, kg/m^3^ (0–4 mm)				Coarse Aggregate (Galala Marble) kg/m^3^ (<10 mm)	W/C
			Sand	Bulk-Bi_2_O_3_	Nano-Bi_2_O_3_	Bi_2_O_3_ wt%		
C-0 Blank	208	417	625	0	0	0	1250	0.50
C-B5	208	417	594	31	0	5		
C-B7	208	417	581	44	0	7		
C-N5	208	417	594	0	31	5		
C-N10	208	417	563	0	63	10		
C-N15	208	417	531	0	94	15		

**Table 2:** Elemental composition and densities of concrete samples.

Elements	C-0	C-B5	C-B7	C-N5	C-N10	C-N15
C	25.44	23.07	22.12	24.49	21.5	20.2
O	46.95	45.5	46.32	47.85	46.05	47.05
Na	0.57	0.33	0.23	0.35	0.2	0.28
Mg	0.77	0.7	0.64	0.77	0.74	0.81
Al	1.96	1.75	1.83	2.51	2.36	2.14
Si	8.73	10.55	11.51	8.73	9.3	8.3
S	0.86	0.82	0.79	0.8	0.81	0.9
Ca	11.47	12.6	11.44	10.02	12.9	11.87
Fe	3.05	3.14	2.97	2.91	3.1	3.9
Bi	0	1.34	1.95	1.37	2.84	4.35
Total	100	100	100	100	100	100
Density, g/cm^3^	2.70	2.74	2.80	2.73	2.75	2.78

**Table 3:** The specifications of radioactive point sources used in the present work with Reference. Date 1 June 2009.

Nuclide	Energy, MeV	Emission Probability	Activity, kBq	Uncertainty
Am-241	0.0595	35.9	259	±2.6
Cs-137	0.662	34.1	385	±4.0
Eu-152	0.122	28.4	290	±4.0
	0.245	26.6		
	0.344	14		
	0.964	20.87		
	1.408	85.21		
Co-60	1.173	99.9	212.1	±1.5
	1.333	99.982		

**Table 4:** The MAC of bulk samples using experimental and theoretical methods and the relative deviation between the experimental and XCOM (**∆_1_%**) as well as the relative deviation between the experimental and Geant4 simulation (**∆_2_%**).

Sample	Energy, MeV	MAC, cm^2^/g			∆_1_%	∆_2_%
		XCOM	EXP	Geant4		
C-0	0.0595	0.3708	0.3754	0.3736	1.23	0.46
	0.662	0.1670	0.1696	0.1710	1.55	−0.85
	0.122	0.1182	0.1194	0.1182	1.01	1.01
	0.245	0.1026	0.1016	0.1018	−0.98	−0.22
	0.344	0.0774	0.0765	0.0760	−1.2	0.65
	0.964	0.0649	0.0657	0.0654	1.22	0.45
	1.408	0.0588	0.0585	0.0586	−0.55	−0.25
	1.173	0.0551	0.0555	0.0550	0.78	0.99
	1.333	0.0536	0.0541	0.0538	0.88	0.42
C-B5	0.0595	0.4440	0.4484	0.4508	−0.97	−0.55
	0.662	0.2126	0.2100	0.2113	1.24	−0.63
	0.122	0.1254	0.1246	0.1235	0.63	0.87
	0.245	0.1055	0.1068	0.1058	−1.25	0.99
	0.344	0.0779	0.0795	0.0799	−1.97	−0.58
	0.964	0.0650	0.0660	0.0665	−1.53	−0.77
	1.408	0.0589	0.0588	0.0582	0.19	1.02
	1.173	0.0552	0.0545	0.0542	1.15	0.54
	1.333	0.0536	0.0539	0.0539	−0.58	0.12
C-B7	0.0595	0.4698	0.4772	0.4762	1.58	0.22
	0.662	0.2325	0.2305	0.2285	−0.87	0.85
	0.122	0.1285	0.1301	0.1295	1.22	0.44
	0.245	0.1068	0.1049	0.1038	−1.82	1.02
	0.344	0.0781	0.0793	0.0797	1.44	−0.58
	0.964	0.0651	0.0656	0.0649	0.77	1.01
	1.408	0.0589	0.0591	0.0598	0.28	−1.15
	1.173	0.0552	0.0557	0.0559	1.024	−0.25
	1.333	0.0536	0.0544	0.0541	1.44	0.55

## Data Availability

Data is contained within the article.
